# Association between Climate Factors and Dengue Fever in Asuncion, Paraguay: A Generalized Additive Model

**DOI:** 10.3390/ijerph191912192

**Published:** 2022-09-26

**Authors:** Raquel Elizabeth Gómez Gómez, Jeehyun Kim, Kwan Hong, Jin Young Jang, Trishna Kisiju, Soojin Kim, Byung Chul Chun

**Affiliations:** 1 Department of Preventive Medicine, College of Medicine, Korea University, Seoul 02841, Korea; 2 Graduate School of Public Health, Korea University, Seoul 02841, Korea; 3 Transdisciplinary Major in Learning Health Systems, Department of Healthcare Sciences, Graduate School, Korea University, Seoul 02841, Korea

**Keywords:** generalized additive model, dengue fever, climate factors, Asuncion, Paraguay

## Abstract

Dengue fever has been endemic in Paraguay since 2009 and is a major cause of public-health-management-related burdens. However, Paraguay still lacks information on the association between climate factors and dengue fever. We aimed to investigate the association between climatic factors and dengue fever in Asuncion. Cumulative dengue cases from January 2014 to December 2020 were extracted weekly, and new cases and incidence rates of dengue fever were calculated. Climate factor data were aggregated weekly, associations between dengue cases and climate factors were analyzed, and variables were selected to construct our model. A generalized additive model was used, and the best model was selected based on Akaike information criteria. Piecewise regression analyses were performed for non-linear climate factors. Wind and relative humidity were negatively associated with dengue cases, and minimum temperature was positively associated with dengue cases when the temperature was less than 21.3 °C and negatively associated with dengue when greater than 21.3 °C. Additional studies on dengue fever in Asuncion and other cities are needed to better understand dengue fever.

## 1. Introduction

The re-emergence of arthropod-borne diseases and the expansion of vectors out of natural tropical areas are increasing and receiving attention from both public health officers and veterinarians [1].

Dengue fever is caused by an RNA virus belonging to the family Flaviviridae. It has four serotypes (DENV-1, DENV-2, DENV-3, and DENV-4) and it is transmitted by the bites of infected mosquitoes. Approximately 300 species of mosquitoes are of medical importance, but *Aedes* spp. (*Aedes aegypti* and *Aedes albopictus*) are responsible for transmitting dengue fever and other infectious diseases [2,3].

There has been an increase in the transmission of dengue, and many countries are hyperendemic for all four serotypes. The increase in infections and risk of dengue is due to a number of different factors, including the expansion of mosquito habitats, the adaptation of mosquitoes, environmental factors, social factors, population growth, poor urban planning, and the circulation of more than one serotype [4]. According to the World Health Organization (WHO), the most affected regions include Southeast Asia, the Western Pacific, and the Americas, and it is common in more than 100 countries. In 2019, the Americas reported 3.1 million cases, and this number continued to increase in 2020 and 2021 in Brazil, Ecuador, Peru, Colombia, and Paraguay [5]. Moreover, according to the Center for Disease Control and Prevention (CDC), the estimated number of people infected with the dengue virus is approximately 400 million, and about four billion people live in areas at risk of dengue [6].

Before 1988, Paraguay had not experienced dengue fever [7]. However, the introduction of DENV-1 caused an outbreak of more than 42,000 cases from 1988–1989, affecting mainly the city of Asuncion and surrounding areas. Dengue fever has been endemic in Paraguay since 2009, and from 2011 to 2013, the country suffered continuous dengue epidemics with all four dengue serotypes [8].

Therefore, our study aims to evaluate the association between climate factors and dengue in Asuncion, Paraguay. Understanding the interaction between the dengue vector and Asuncion’s climate factors can help enlighten useful information to do better predictions as well as improve the dengue control system in Paraguay.

## 2. Materials and Methods

### 2.1. Study Area

Paraguay is a landlocked country bordered by Bolivia, Brazil, and Argentina. Asuncion (25°16′5″ S, 57°38′5″ W) has an area of 117 km^2^. The capital city is located on the left bank of the Paraguay River, which divides the country into two large regions. Asuncion is surrounded by the Central department, which borders it to the north, east, and south [9]. The climate of Paraguay is tropical–subtropical, varying between humid mesothermal and semi-arid megathermal with the general characteristics of a continental climate [10]. According to the Köppen–Geiger climate classification, Asuncion is classified as Cfa (warm temperate climate, fully humid with hot summers). Moreover, temperatures can vary drastically, resulting in frost (winter) and heat waves (summer) [11].

### 2.2. Dengue Data and Climate Factor Data

For this study, we extracted data from the weekly bulletin of the General Directorate of Health Surveillance (D.G.V.S.) of the Ministry of Public Health and Social Welfare. Weekly data from January 2014 to December 2020 were collected. New cases were reported weekly, and the incidence was calculated as the number of new cases divided by the mid-year population for each year. The population in Asuncion per year was retrieved from the National Institute of Statistics (INE).

Asuncion’s climate data are not accessible to the public and must be requested from the Directorate of Meteorology and Hydrology. The application form was downloaded from the website, and climatological data from January 2013–December 2020 were requested as follows: rainfall (mm), sunshine (h), minimum temperature (°Celsius), maximum temperature (°Celsius), mean temperature (°Celsius), atmospheric pressure (hectopascal), relative humidity (percentage), cloudiness (oktas), and wind (kilometers per hour). We received climate data as daily data and aggregated it as weekly data to match the dengue fever surveillance data.

### 2.3. Statistical Analysis

A time-series analysis was conducted to evaluate the trend and seasonality of dengue cases and each climate factor, and then a correlation analysis was conducted to evaluate the relationship between dengue cases and each weather variable. Univariate analyses were performed to evaluate the association between the dependent variable (dengue cases) and the independent variables (climate factors).

The association between dengue cases and climatic factors was also analyzed. Before building the model, lags of 0–10 for each variable were calculated. The criteria for selecting the length of the lag were based on previous literature, and the effect of the climate variables on dengue fever may have required an induction period of many weeks, such as the development period required for mosquito eggs to become adults, the transmission processes, and sexual maturation of the vector. Then, weather variables with their corresponding lags were selected according to the rho of Spearman’s test. The generalized additive model (GAM) was used to evaluate the statistical association between climate factors and dengue cases with a negative binomial and adjusted for seasonality and a logarithmic link function.

One of the challenges in analyzing climatic data is that they are generally non-normally distributed, which is why the application of a generalized linear model (GLM) fails when it comes to analyzing their effects in real life. Therefore, GAM, the model developed by Hastie and Tibshirani in 1990, is a developed mathematical model based on the GLM. GAM is a generalized linear model that provides the flexibility of analyzing non-parametric data with a linear predictor linking the sum of smooth functions of covariates [12,13].

The basic model was as follows:

$$\begin{array}{ll} \left[E(\mathrm{Yt})\right] & =\alpha +s(\mathrm{average}\;\mathrm{humidity})+s(\mathrm{average}\;\mathrm{pressure})+s(\mathrm{minimum}\;\mathrm{temperature})\\ & +\mathrm{average}\;\mathrm{wind}+\mathrm{average}\;\mathrm{sunshine}+\mathrm{average}\;\mathrm{rainfall}+\mathrm{time}+\sin\frac{2\pi t}{T}\\ & +\cos\frac{2\pi t}{T}+\mathrm{off}(\log[\mathrm{population}]) \end{array}$$

where E(Yt) is the expected number of dengue cases per epidemiological week, α is the intercept, s is the spline function, t is the time, sine and cosine are the functions of seasonality, and T represents 365 days. The variables rainfall, wind, and sunshine were included without a spline function as they presented a degree of freedom of 1.

The best model was selected based on the Akaike information criterion (AIC). After selecting the final model, the incidence rate ratio (IRR) was calculated for each variable in relation to dengue cases during the study period. Piecewise linear regression analyses were performed for variables that did not exhibit a linear association. As the data did not follow a linear trend and their interpretation may have been difficult, piecewise linear regression analyses allowed for modeling the regression into “pieces”.

A model for piecewise linear regression was developed based on our selected model, and the variable to be cut was specified. A breakpoint was considered for the selected variable where the RR for the variable was equal to one. Then, the IRR for each variable segment was calculated. Statistical analyses were performed in the R software version 4.0.1 (RStudio, Boston, MA, USA).

## 3. Results

**Figure 1:**
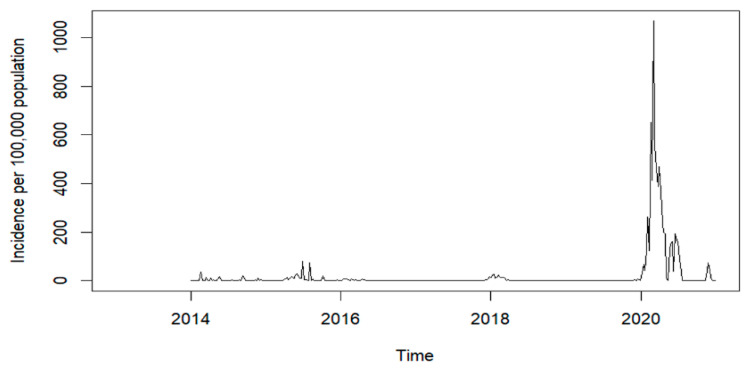
Total number of cases per year from 2014 to 2020 in Asuncion, Paraguay.

During the study period, 40,593 cases of dengue fever were observed in Asuncion. The highest number of cases was reported in 2020 with 35,919 cases (incidence 6964.85 per 100,000 population), followed by 2015 with 1933 reported cases (incidence 412.13 per 100,000 population). The lowest number of cases was reported in 2019 with 174 cases (incidence 33.3 per 100,000 population), followed by 233 cases in 2017 (incidence 45.95 per 100,000 population) (Figure 1). The dengue data are summarized in Table 1.

**Figure 2:**
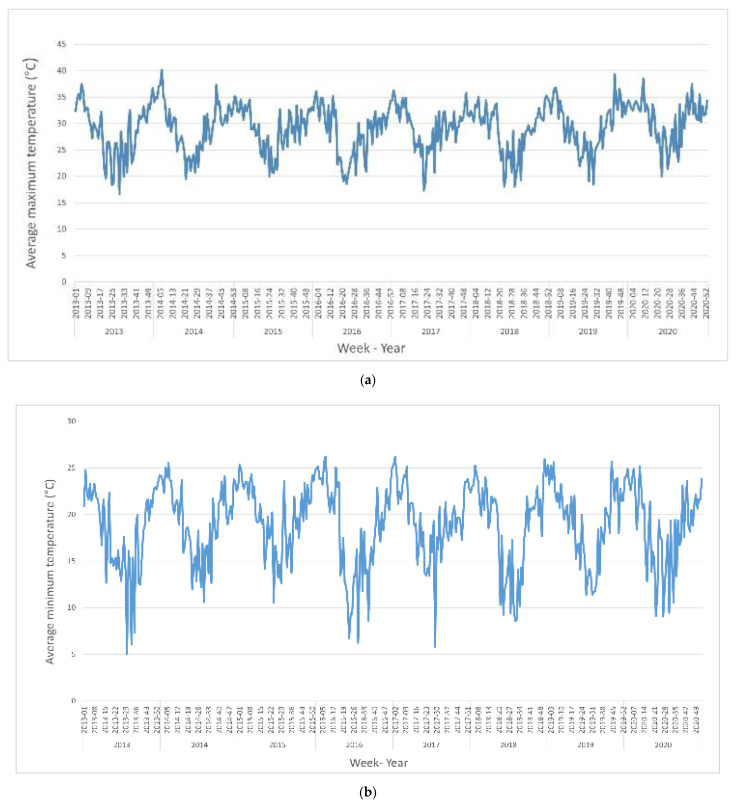

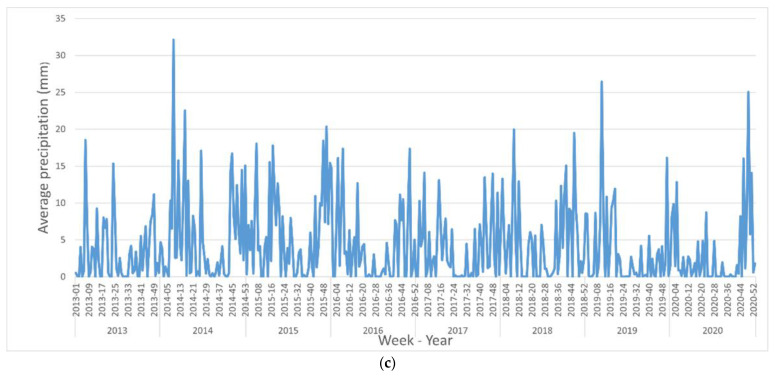
(**a**) Weekly average maximum temperature, (**b**) weekly average minimum temperature, and (**c**) average precipitation in Asuncion, Paraguay, 2013–2020.

During the study period, the average minimum temperature was 18.89 °C (range 5.06–26.23 °C), and the lowest minimum temperatures were between June and August. The average maximum temperature was 29.18 °C (range 16.69–32.54 °C), and the hottest months of the year were November, December, and January. The average precipitation was 4.04 mL (range 0.0–32.13 mL) (Figure 2). Additional climatic variables are summarized in Table 2.

The environmental variables were minimum temperature at a lag of 10 weeks, rainfall at a lag of 10 weeks, relative pressure at a lag of 10 weeks, sunshine at a lag of 8 weeks, wind at a lag of 4 weeks, and humidity at a lag of 1 week (Table 3). The results of the IRR between dengue cases and climatic factors are shown in Table 4.

**Figure 3:**
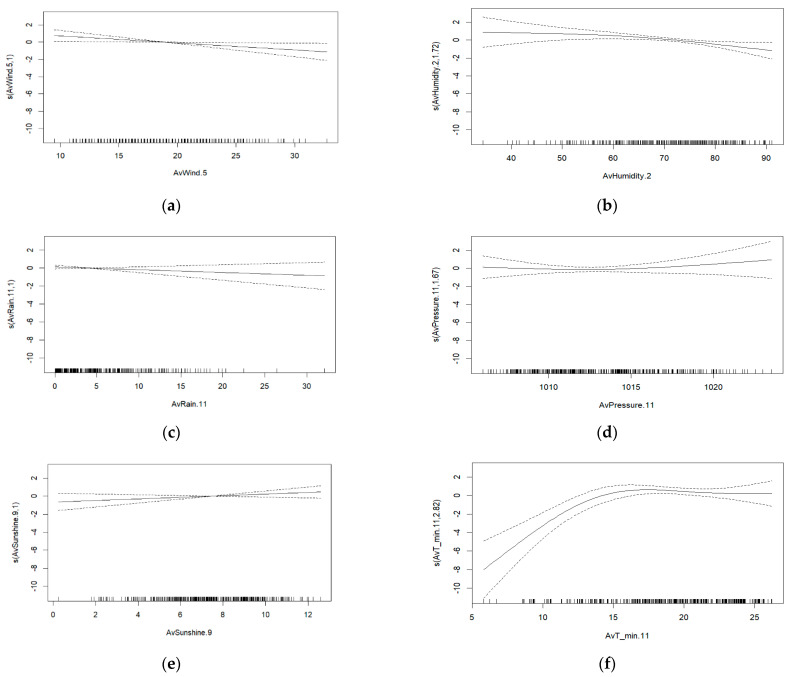
Association between dengue fever cases and (**a**) wind, (**b**) relative humidity, (**c**) atmospheric pressure, (**d**) precipitation, (**e**) sunshine, and (**f**) minimum temperature during 2014–2020, Asuncion, Paraguay.

Wind had a significant inverse association with dengue cases, with an IRR of 0.922 (95% CI 0.86–0.99). There was also a significant inverse association between dengue cases and relative humidity (IRR 0.956 (95% CI 0.93–0.99)). The association between atmospheric pressure and dengue cases was positive with an IRR of 1.045 (95% CI 0.80–1.23). For rainfall, the association with dengue cases was negative, with an IRR of 0.960 (95% CI 0.92–1.02). Sunshine was positively associated with dengue cases, with an IRR of 1.094 (95% CI 0.958–1.249) (Figure 3).

In the case of the minimum temperature before calculating the IRR, piecewise linear regression was conducted, because this variable followed a different linear trend. The breakpoint value was found at the temperature of 21.3 °C. A significant association was found between the average minimum temperature and dengue cases. That is, when the minimum temperature increased by more than 21.3 °C, dengue cases decreased (95% CI 0.47–0.95) (Table 5).

## 4. Discussion

This study aimed to determine the relationship between dengue fever and climatic factors in Asuncion between January 2014 and December 2020. Many studies have analyzed the association between dengue incidence and climatic factors [14,15,16]. However, Paraguay still lacks information on the association between dengue fever and other variables, such as environmental variables. Evaluating the relationship for a better understanding of the vector and climate in each endemic zone, such as Asuncion, is crucial.

The impact of rainfall on mosquito growth and distribution varies with the geographic location under study. For instance, in a study in Thailand, both positive and negative associations between dengue fever and precipitation were observed, depending on the provinces [17]. Our results showed a negative association between dengue cases and rainfall. This could be due to the fact that the geography of Paraguay consists of grassy plains and low hills. Thus, the formation of stagnant pools after rainfall in the plain of Paraguay may make it less likely for mosquitoes to breed. In addition, some studies have suggested that heavy rainfall can cause an overflow of mosquito eggs and larvae, which may be related to the negative association between variable precipitation and dengue cases [18,19].

Moreover, a study by Yuan et al., also found a negative association between rain and dengue but also suggested that the pre-monsoon season can increase the incidence when the intensity of rain is lower [19]. In addition, a study in Barbados on the rain and dengue cases found a weak association between dengue and rainfall [20].

The variable of sunshine demonstrated a positive association with dengue cases in agreement with dengue studies conducted in Sri Lanka [16] and Vietnam [21,22] and a Malaria study in southern China [23]. This could be connected to the beginning of the end of the mosquito’s diapause, which is marked by a photoperiod [3]. This is combined with Paraguay’s twice-a-year “daylight-saving time”, which consists of a delay of one hour with the aim of increasing the use of sunlight. This daylight-saving time could be related to the increase in outdoor activities during the spring–summer season, such as going to parks, going near the river, and increasing exercise. Therefore, the increase in these types of outdoor activities creates more opportunities for mosquitoes to detect and be in contact with their host.

In relation to wind, we found an inverse association with dengue cases, with similar results found in Barbados [20], Ecuador [24], and Guangzhou, China [25]. Wind can help with the dispersion of the vector; however, it can also affect the flight of adult mosquitoes [26]. Wind can also accelerate vaporization, which could possibly lead to a decrease in areas where female mosquitoes can oviposit. A study in Ecuador found that an increase in winds resulted in a decrease in the number of dengue cases, suggesting that wind can reduce the density of mosquito in an area and consequently reduce the probability of being bitten [24].

In this study, we found that relative humidity was negatively associated with dengue cases. Asuncion is humid throughout the year, even during the dry season, which is likely one of the reasons for this result. Similar findings were found in a study in Brazil, where humidity was high, even during the non-monsoon season [27]. In addition, it has been suggested that humidity levels between 60–70% shorten the lifespan of mosquitoes. Our findings suggest a positive association between atmospheric pressure and dengue fever. Humidity and pressure have an inverse relationship, supporting our finding that atmospheric pressure had a positive association with dengue compared to humidity.

Additionally, our results showed that a minimum temperature under 21.3 °C was related to an increase in dengue cases. This result may be due to the increase in outdoor activities and the favorable climate. A study suggested that the optimal temperature for a mosquito’s flight was 21 °C, and it also was suggested that, at around 15 °C, mosquitoes fly farther [28]. Additionally, another study suggested that, when the temperature was less than 21 °C, the mosquito life expectancy could be longer [29].

In contrast, minimum temperatures above 21.3 °C, were related to a decrease in dengue cases. A study related to the Zika virus demonstrated that an increase in temperature can affect the vectorial capacity of Aedes mosquitoes [30]. This minimum temperature value could be important as one of the factors to take into consideration to predict a dengue outbreak in Asuncion city.

Our results were compared with the sensitivity analyses, which excluded the data from 2020, that aimed to assess whether our results were due to climatic factors only or if other factors had any influence, particularly for the 2020 outbreak. We found that the climatic factors were comparable to our results which include the 2020 data. Among these temperatures, the minimum temperature was found to be significant for dengue fever in Asuncion. Rainfall, wind, atmospheric pressure, and humidity were significant climatic factors to dengue. Factors that showed differences between the models that include and exclude the 2020 data were cloudiness and sunshine. We can infer that these two variables did not have significant influence on dengue fever in Asuncion.

However, the 2020 outbreak may also have been affected either by climate factors or non-environmental factors, including social factors, such as the contamination of the streams that cross the city. According to the Ministry of Health in 2020, most reported cases of dengue in Asuncion were from neighborhoods near streams.

Indeed, most of the cases reported during the dengue outbreak in 2020 were between December and March. However, dengue cases are constantly reported; therefore, the strategy used by the dengue surveillance system is constant throughout the year. There is a suggestion that the disruption and reduction in the activities of the dengue control programs during the COVID-19 pandemic could affect indirectly dengue fever. However, this hypothesis needs to be analyzed in future studies.

Our study had some limitations. First, the validity and completeness of the surveillance system for dengue fever remain unknown. Second, dengue cases and other variables such as health behavior, sex, and age were not included. Third, this study was limited to a single city. Fourth, ecological niche modeling as a correlative approach to understanding potential habitats to the host could not be used because of the unavailability of mosquito data Some strengths of our study include that this was the first analysis of dengue fever that considered the climatic factors in Asuncion. Second, GAM was used to analyze our data and improve our understanding of dengue fever in Asuncion. Third, the results can help recognize some of the dynamics of dengue and climate factors in Asuncion, Paraguay.

## 5. Conclusions

In our study, we found an association between climatic factors and dengue fever in Asuncion. Wind and relative humidity were negatively associated with dengue cases. Below 21.3 °C, minimum temperature showed an association with dengue fever cases that was positive, and a negative association was shown when minimum temperature increased to more than 21.3 °C. Our results showed similarities and discrepancies with those of other studies related to dengue fever and environmental factors. It could be inferred that each region, area, and country is affected in different ways by their characteristics and local climate, which, in turn, influences the development of transmitted diseases by vectors.

To help improve the knowledge and a better understanding of dengue fever and its vectors, further studies should be conducted on dengue fever in Asuncion and other cities. In addition, we suggest the utilization of other regional indexes, such as socio-economic indexes. This study found valuable information that can be used for the effective vector control of dengue by considering climate factors.

## Figures and Tables

**Table 1:** Summary statistics of weekly dengue fever data from 2014 to 2020 in Asuncion, Paraguay.

Dengue Data	Minimum	Mean	Maximum	1Q	Median	3Q
Cumulative dengue data	0	4122	35,919	37	354	1001
New cases of dengue	0.0	110.9	5564	0.0	2.0	14.0
Incidence per 100,000 population	0.00	21.623	1066.8	1010.0	0.380	3.857

**Table 2:** Summary statistics of weekly climate factors from 2013 to 2020 in Asuncion, Paraguay.

Climate Variables	Minimum	Mean (SD)	Maximum	1Q	Median	3Q
Average Rainfall (mm)	0.00	4.04 (5.28)	32.13	0.06	1.79	6.58
Average Wind (km/h)	8.70	19.3 (4.39)	34.09	16.28	19.02	22.17
Average Pressure (hPa)	1006.0	1013 (3.69)	1025.0	1010.0	1012.0	1015.0
Average Sunshine (h)	0.23	7.47 (2.31)	13.04	5.98	7.54	9.14
Average relative humidity (%)	34.43	70.41 (9.97)	91.71	64.61	71.64	77.39
Average Cloudiness (oktas)	0.14	3.93 (1.46)	7.86	2.86	4.00	4.86
Average minimum temperature (°C)	5.06	18.89 (4.37)	26.23	16.26	19.51	22.33
Average mean temperature (°C)	10.30	23.31 (4.32)	32.33	20.19	21.14	26.60
Average maximum temperature (°C)	16.69	29.18 (4.55)	40.09	26.21	30.03	35.54

SD, standard deviation; 1Q, first quantile; 3Q, third quantile.

**Table 3:** Summary of the cross-correlation analysis, Asuncion, Paraguay.

Climate Variable	Lag (Week)	rho	*p* Value
Average rainfall (mm)	10	0.189	<0.001
Average wind (km/h)	4	−0.188	<0.001
Average sunshine (h)	8	0.198	0.001
Average humidity (%)	1	0.169	0.001
Average pressure (hPa)	10	−0.316	<0.001
Average minimum temperature (°C)	10	0.335	<0.001

**Table 4:** Results of the incidence rate ratio between climatic factors and dengue cases, Asuncion, Paraguay.

Climate Variables	IRR ^†^	Percentage (%)	95% CI ^‡^
Average rainfall (mm)	0.960	3.05	0.918–1.024
Average wind (km/h)	0.922	7.81	0.859–0.980 *
Average sunshine (h)	1.094	9.37	0.958–1.249
Average relative humidity (%)	0.956	4.40	0.927–0.986 *
Average pressure (hPa)	1.045	4.47	0.897–1.217
Average minimum temperature (°C)	1.11	11.1	0.969–1.272

* *p* value < 0.05; ^†^ IRR: Incidence rate ratio, ^‡^ 95% CI: confidence interval.

**Table 5:** Results of the piecewise analysis and incidence rate ratio between minimum temperature and dengue cases.

Climate Variable	IRR ^†^	95% CI ^‡^ (*p* Value)
Average minimum temperature < 21.3 °C	1.164	0.978–1.385 (0.080)
Average minimum temperature > 21.3 °C	0.671	0.473–0.951 (0.022) *

* *p* value < 0.05; ^†^ IRR: Incidence rate ratio, ^‡^ 95% CI: confidence interval.

## Data Availability

Climate data were obtained from the Directorate of Meteorology and Hydrology (DINAC). Due to legal restrictions, the database cannot be made publicly available. However, the data are available from the authors upon reasonable request and permission. The dengue data can be downloaded through proper procedures from https://dgvs.mspbs.gov.py/, accessed on 9 August 2022.
